# Domoic Acid - A New Toxin in the Croatian Adriatic Shellfish Toxin Profile

**DOI:** 10.3390/molecules15106835

**Published:** 2010-10-08

**Authors:** Ivana Ujević, Živana Ninčević-Gladan, Romana Roje, Sanda Skejić, Jasna Arapov, Ivona Marasović

**Affiliations:** 1 Institute of Oceanography and Fisheries, Šetalište I. Meštrovića 63, 21000 Split, Croatia P.O. Box 500, Croatia; 2 The Erasmus Mundus Master of Science in Marine Biodiversity and Conservation, Doverska 9, 21000 Split, Croatia

**Keywords:** Amnesic shellfish poisoning (ASP), retention of domoic acid, mussels, *Pseudo-nitzschia*, Adriatic Sea

## Abstract

This is the first study that presents concentrations of domoic acid detected in the whole shellfish tissue from breeding and harvesting areas along the Croatian coast of the Adriatic Sea during the period 2006 to 2008. Shellfish sample analyses after SAX cleaning procedures, using a UV-DAD-HPLC system, showed the presence of domoic acid in four species. The most prevalent of those species were the blue mussel (*Mytilus galloprovincialis*), followed by European flat oyster (*Ostrea edulis*), Mediterranean scallop(*Pecten jacobaeus*) and proteus scallop (*Flexopecten proteus*). Domoic acid, a potentially lethal phycotoxin that causes amnesic shellfish poisoning (ASP), was detected for the first time in January 2006 with the highest value of 6.5486 μg g^-1 ^in whole shellfish tissue. *Pseudo-nitzschia* spp. bloom events preceded these high domoic acid concentrations. According to this study, retention of domoic acid in the blue mussel *M. galloprovincialis* is more than 42 days. This investigation indicates the first presence of domoic acid in Croatian shellfish, but in concentrations under the regulatory limit (20 μg g^-1^), therefore shellfish consumption was not found to endanger human health.

## 1. Introduction

Occurrence of harmful algal species in the marine environment is a well known cause of human poisoning, mostly by the consumption of contaminated marine products such as shellfish. The seriousness of the threat of amnesic shellfish poisoning (ASP) has been recognized after a case of poisoning in Canada in 1987 [1] when a toxic episode caused the death of at least three people and more than a hundred exhibited neurological problems after consuming blue mussels (*Mytilus edulis)* from Prince Edward Island contaminated with domoic acid (DA) at levels up to 790 µg g^-1^ [2]. Domoic acid production was linked to the diatom *Pseudo-nitzschia multiseries* and thus this species was identified as a causative organism of the poisoning [3]. This species was also found to have been involved in DA accumulation in French shellfish [4]. After that case, ASP research in the area expanded, with DA subsequently being found in relation to other *Pseudo-nitzschia* species, including *Pseudo-nitzschia australis* [5,6,7], *P. pseudodelicatissima* [8], *P. pungens* [9], *P. seriata* [10], *P. calliantha* [11], *P. multiseries* [12] and *P. fraudulenta* [5,6,11]. In addition to the above marine diatoms of the genus *Pseudo-nitzschia*, known producers of DA are red algae of the genus *Chondria* [13,14].

Domoic acid is a non-protein, crystalline, water soluble, cyclic amino acid that binds irreversibly to glutamate receptor sites, causing destructive neuronal depolarization and permanent short-term memory loss in mammals [15]. Apart from the neurotoxic properties of DA, genotoxic effects on fish have been established experimentally. Domoic acid induces the formation of micronuclei and DNA strand breaks in peripheral erythrocytes [16]. There are currently ten known isomers of DA, including the isodomoic acids A through H and the domoic acid 5’ diastereomer [17]. Recent research suggests that isodomoic acid A, B and C pose a lower risk to human health than DA itself [18]. 

Domoic acid accumulates in filter-feeding shellfish by consuming DA producing phytoplankton. In Europe, reports of DA in wild or cultivated shellfish have included the coast of Portugal [19], as well as the Mediterranean regions of France [4], Italy [20] and Greece [21]. James *et al*. conducted a study, following the first discovery of DA in Ireland in December 1999, and determined the extent of DA contamination of four bivalve species shellfish [22]. DA concentrations in king scallop and variability both within and between different harvesting grounds from around the Isle of Man were reported by Bogan *et al*. [23]. During 1996, an ASP toxic episode affected Galician (northwest Spain) scallops (*Pecten maximus*) [24].

Trophic transfer of DA is possible by vectors other than shellfish such as copepods, krill [25,26], tunicates [27], cephalopods, cuttlefish [28], planktivorous fish [6,19], and top predators such as sperm whales [7], seabirds [29] and sea lions [30,31]. Fish are very potent vectors because of a lack of acute symptomology during toxic blooms which allows fish to continue feeding and to continue to accumulate and pass on the toxin through the food web to susceptible organisms [32]. Following the Canadian toxic outbreak, DA has been detected in a variety of shellfish throughout the world [4,19,21,33]. Ciminello *et al.* [20] collected mussels from the Italian western part of the Adriatic Sea during the period 2000 to 2004, analysed the samples by HILIC/MS and recorded the presence of DA as a new toxin that entered the mussel (*M. galloprovincialis*) toxin profile in December 2000 (2.5 μg g^-1^).

Investigation of DA toxicity in Croatian waters has started to ensure the safety of shellfish for human consumption. According to Regulation (EC) 853/2004 [34] and Croatian legislation, the maximum permitted level for DA in shellfish edible parts is 20 µg g^-1^. The purpose of this paper is to establish the temporal and spatial distribution of DA in different species of shellfish of the Adriatic Sea, and the occurrence of DA compared with the level of *Pseudo-nitzschia* spp. abundance in seawater.

## 2. Results and Discussion

A total number of 1,407 shellfish samples and corresponding seawater samples collected from Croatian breeding and harvesting areas were analyzed from 2006 until 2008. Area of investigation comprise of 22 shellfish breeding and 4 harvesting areas (ZI1, ZI2, ZI3 and SI4) in three zones: North Adriatic (ZI1, ZI2, ZI3, SS1, SV1, LZ1, ME1, UB1 and RZ1), Central Adriatic (SI1 – SI5) and South Adriatic (MZ1 – MZ7 and US1). Means and ranges of temperature measured at each farming area during this three year period are given in Table 1 and the salinity is given in Table 2. 

**Table 1:** Surface and bottom temperature at the investigated stations for 2006 – 2008 period.

Research area of the Adriatic Sea		Mean	Min.	Max.	Std.dev.
**Surface**	**North**	17.3	7.6	26.7	5.8
	**Central**	16.8	5.4	27.1	6.1
	**South**	17.8	6.5	27.3	6.0
**Bottom**	**North**	16.7	8.1	24.9	5.2
	**Central**	17.4	12.0	22.1	2.9
	**South**	17.6	6.7	25.4	4.5

**Table 2:** Surface and bottom salinity at the investigated stations for 2006 – 2008 period.

Research area of the Adriatic Sea		Mean	Min.	Max.	Std.dev.
**Surface**	**North**	33.5	3.7	37.7	7.1
	**Central**	15.2	1.9	38.3	9.1
	**South**	33.7	24.2	37.9	2.8
**Bottom**	**North**	36.6	28.8	37.9	1.4
	**Central**	36.5	32.4	38.2	1.3
	**South**	36.2	29.8	37.9	1.2

**Figure 1:**
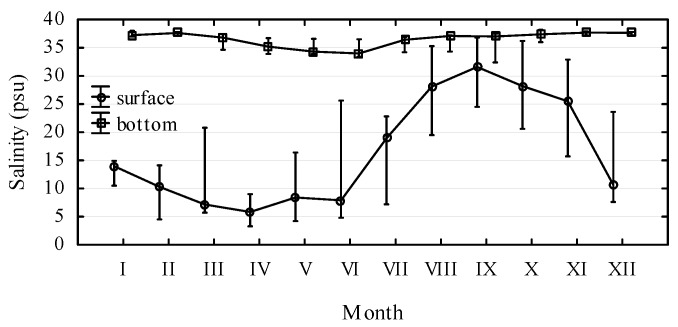
Difference between the surface and the bottom (10 m) salinity at central Adriatic Sea stations SI1 – SI5 with mean monthly values; bars indicate min. and max. values for the 3-year investigation period (2006–2008).

The Central Adriatic Sea samples showed a noticeable difference in salinity between the surface and the bottom water layers (Figure 1). The bottom salinity ranged from 32 to 37 psu, while the surface layer salinity ranged from almost pure fresh water of 1 to 36 psu. Temperature ranged from 5.4 ºC to 27.1 ºC in February and August, respectively. 

**Figure 2:**
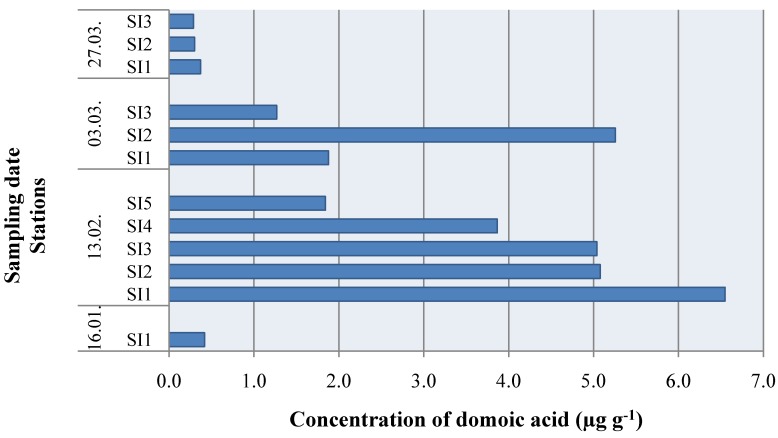
Domoic acid in shellfish samples (*Mytilus galloprovincialis*) from the central Adriatic Sea in 2006 (see Figure 1).

During the period of investigation, DA was detected for the first time in this area on the 16th January 2006 in a mussel sample from SI1, and its presence was also evident through February and March. A maximum measured concentration of 6.5486 μg g^-1^ was detected in February at SI1 and decreased towards SI5 as the influence of fresh water diminished (Figure 2). 

**Figure 3:**
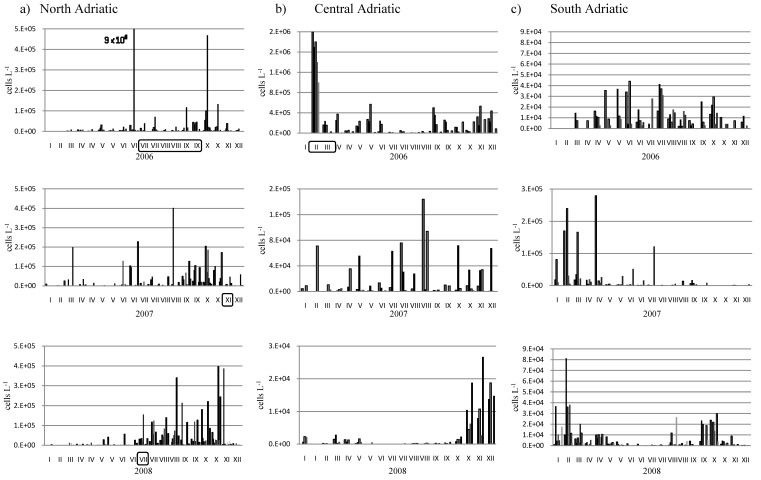
Annual variability of *Pseudo-nitschia* spp. number of cells from North Adriatic (a), Central Adriatic (b) and South Adriatic(c). Periods of the DA occurrence are framed.

**Figure 4:**
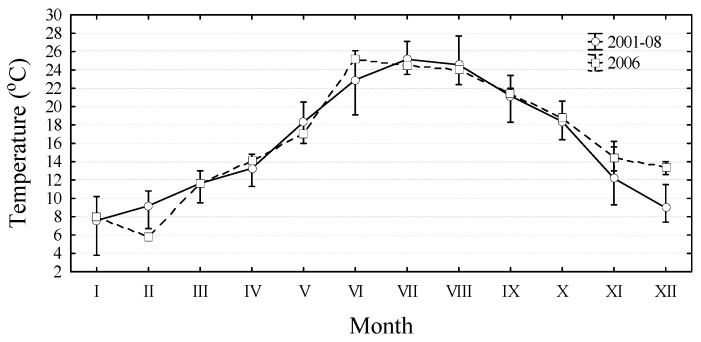
Mean monthly temperatures with the ranges during 2001 to 2008 period (excluding 2006) and in 2006 at central Adriatic Sea stations SI1-SI3.

During March the influence of fresh water became more significant at farms located further downstream, therefore the highest concentrations were in the SI2 samples (Figure 2). All measured concentrations of DA were below the regulatory level of 20 µg g^-1^. The accumulation of DA in mussels was the result of a *Pseudo-nitzschia* spp. bloom (>10^6 ^cells L^-1^) that occurred in February 2006 after a rainfall event (Figure 3b). Although relatively high sea temperatures (14–17 ºC) tend to be associated with increased abundance of *Pseudo-nitzschia* according to Walz *et al*. [35], this study shows that in the central Adriatic Sea, *Pseudo-nitzschia* spp. blooms have occurred in, for this species, extremely cold waters (Figure 4). Namely, in February 2006, the lowest measured temperatures during the eight-year period were found, with a monthly mean of 6 ºC.

Furthermore, this occurrence of contamination of mussel cultivated along the shoreline reveals that 94% of domoic acid content was eliminated from the blue mussel tissue within 42 days (period from the 16th of January to the 27th of March), while their detoxication lasted for 55 days (period from the 16th of January until the 9th April, when the toxin was not longer detected). DA was not detected during 2007 and 2008 in the central Adriatic Sea and *Pseudo-nitzschia* spp. abundances were below 1.0 × 10^5^ cells L^-1^ and 2.0 × 10^4^ cells L^-1^, respectively (Figure 3b).

The maximum abundances of *Pseudo-nitzschia* spp. in the central Adriatic Sea were already found during mid-winter (February) in 1999, when it contributed up to 85% of the total diatom abundance. Morphological analysis had shown that the dominating *Pseudo-nitzschia* species corresponded to *P. calliantha* [36]. It was also discovered in samples from the Gulf of Trieste (Italian north-west Adriatic Sea) and Kaštela Bay in the east-central Adriatic Sea [37]. Caroppo *et al*. [38] found increasing abundance of *P. calliantha* (up to 1.48 × 10^5^ cells L^-1^) during winter water conditions in the Italian south-west Adriatic region, while Spatharis *et al*. [39] counted up to 7 × 10^6 ^cells L^-1^ in the inner part of the Mediterranean Kalloni Gulf (Greece) in February 2005.

Phytoplankton biomass and production are highly variable both in space and time [37] and the phytoplankton dynamics in coastal systems are very complex, particularly in the areas affected by freshwater discharge such as the highly stratified Krka Estuary (SI1-SI5)*.* This study confirmed previous observations on the preference of *Pseudo-nitzschia* species for enclosed water bodies with nutrient loading [39]. *Pseudo-nitzschia* spp. was widely distributed across the Adriatic Sea of both warm and cold climate conditions within the phytoplankton community throughout the investigation period. There are seasonal variations in phytoplankton abundances with increasing numbers in early spring and autumn (Figure 3).

**Figure 5:**
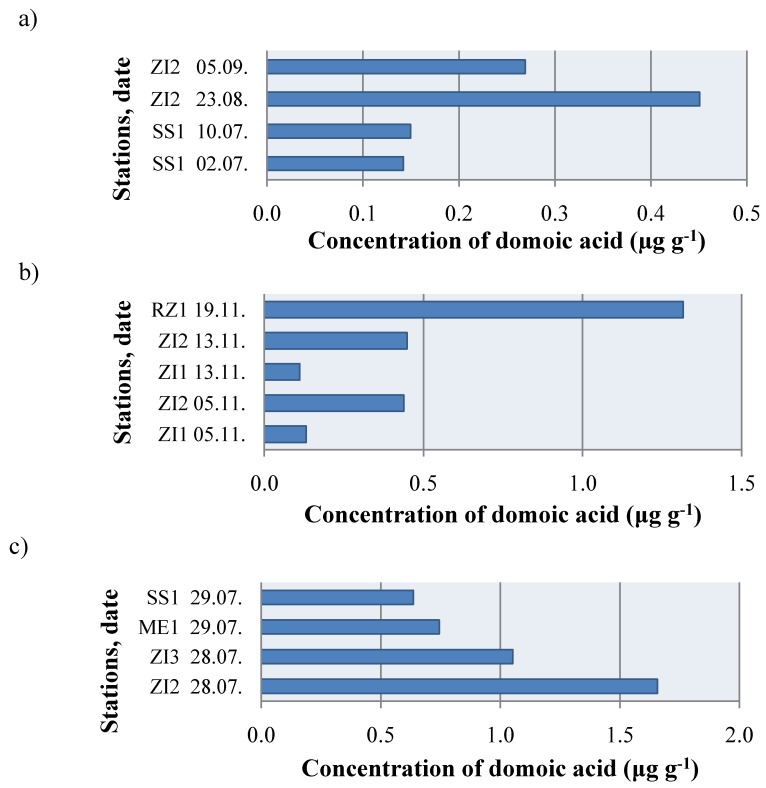
Domoic acid in shellfish samples *Mytilus galloprovincialis* (SS1, RZ1 and ME1)*,* *Flexopecten proteus* (ZI1), *Pecten jacobaeus* (ZI2) and *Ostrea edulis* (ZI3) from the north Adriatic Sea in (a) 2006, (b) 2007 and (c) 2008.

Contaminated samples from the north Adriatic Sea were primarily those of *F. proteus* (ZI1), *P. jacobaeus* (ZI2) and *O. edulis* (ZI3); while the least represented species was the mussel *M. galloprovincialis* (Figure 5). Consequently, although often used as biomarkers, mussels are not necessary good indicator species with respect to ASP contamination. Concentrations of DA were within the range of 0.1117-1.6567 μg g^-1^ (Figure 4). Although DA concentrations were low, a potential risk to human health due to repetitive long-term and low-level exposure calls for continuing investigation of DA [41]; as for the example, Goldstein *et al*. [42] characterized a chronic DA toxicosis syndrome in sea lions that is distinguishable from acute toxicosis.

Toxin was recorded in the north Adriatic Sea after increasing *Pseudo-nitzschia* spp. abundances up to 1.5 × 10^6^ cells L^-1^ for 2006, 2.0 × 10^5^ for 2007 and 1.5 × 10^5^ for 2008 (Figure 3a). *Pseudo-nitzschia calliantha* was found in seven shellfish areas throughout the northern Adriatic Sea as the dominant diatom species in the summer and autumn of 2007 [37]. *Pseudo-nitzschia* spp. were always present throughout the study period in the south Adriatic Sea, although with abundances mostly lower than at the other parts of the investigated area (Figure 3c), and with no DA detected.

It should be taken into considering that not all *Pseudo-nitzschia* species are toxic and that even toxic ones do not always express toxicity. Nevertheless, according to these extensive data on the *Pseudo-nitzschia* spp. abundances in the study area of the Adriatic Sea, we can assume that if the abundance does not reach 1.0 × 10^5^ cells L^-1 ^then the area can be considered safe with respect to ASP. If the abundance exceeds 1.0 × 10^6^ cells L^-1 ^it might indicate the possible occurrence of ASP contamination. These results add to the previously published observations of domoic acid occurrence in shellfish found in many maritime countries of the European Union. 

## 3. Experimental

### 3.1. Collection of shellfish samples

In 2000, Croatia initiated a monitoring program of its shellfish breeding and harvesting areas in accordance with EU Directive 79/923/EEC [43] and EU Directive 91/492/EEC [44]. It includes analysis of shellfish for the presence of algal toxins (for ASP, DSP, and PSP) and seawater for the presence of toxic phytoplankton species.

**Figure 6:**
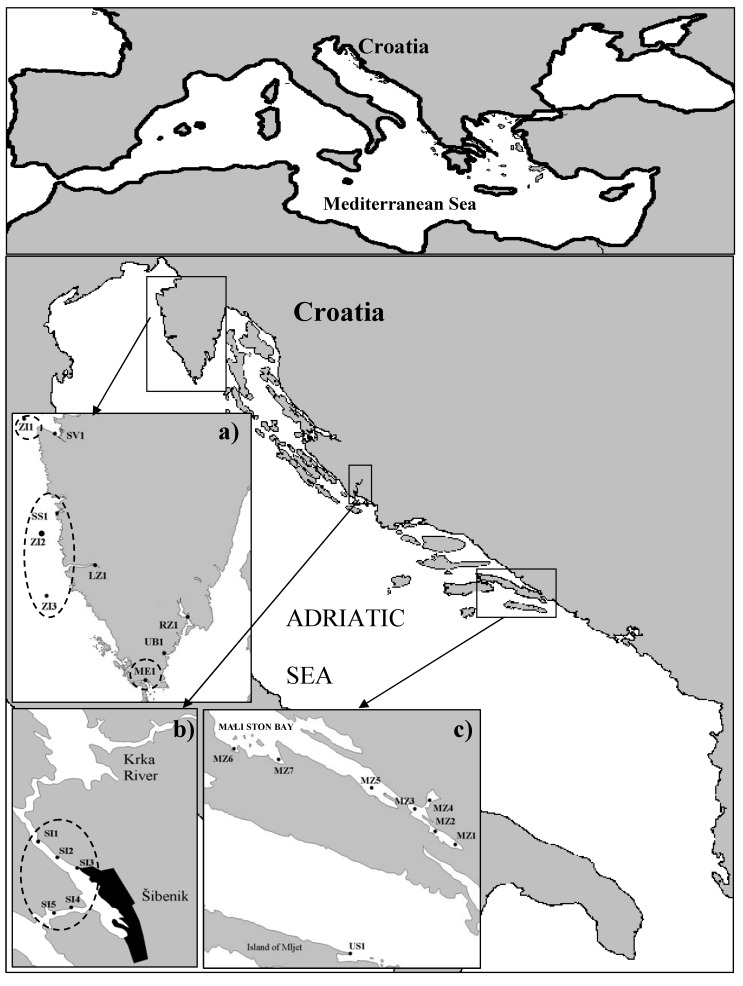
Area of DA and phytoplankton community investigation along Croatian Adriatic coast comprise of 22 shellfish breeding and 4 harvesting areas (ZI1, ZI2, ZI3 and SI4) in three zones: North Adriatic (a), Central Adriatic (b) and South Adriatic (c). Stations where domoic acid was appeared are framed with dashed line.

Domoic acid was investigated in the Croatian most important cultivated bivalve species, *M. galloprovincialis,* from both breeding farms and wild populations of *Flexopecten proteus* (ZI1), *Pecten jacobaeus* (ZI2), *Ostrea edulis* (ZI3) and *Mytilus galloprovincialis* (SI4) during 2006 until 2008 (Figure 6). 

The investigated area comprised of three different farming zones along the eastern Adriatic coast (Figure 6) where the climate is characterized by hot and dry summers (through May to September) and mild and rainy winters. The northern Adriatic breeding farms are located along the coast of the Istrian Peninsula (Figure 6). The central Adriatic breeding and harvesting farms are located in the Šibenik area (Figure 6) influenced by the Krka River Estuary, one of the most productive zones along the Croatian Adriatic coast with fresh water in the surface layer throughout the entire area. This locality is characterized by reduced water exchange with the outer bay area and high nutrient concentrations originating from anthropogenic pollution [45]. The southern Adriatic breeding and harvesting farms are located in the Mali Ston Bay, except for US1 which is located in the aquatorium of Mljet Island (Figure 6). Mali Ston Bay is the most important shellfish farming area in Croatia, with more than one hundred years of tradition.

### 3.2. Preparation of shellfish samples

The method used for ASP toxins, DA and *epi*-DA determination followed the protocol proposed by Quilliam *et al*. [46]. For representative samples, weights of about 100.00 g of soft tissue were used. Homogenized samples (approximately 4.0 g) were extracted with MeOH/H_2_O (1:1, 16 mL) at 10,000 rpm for 5 min. After 40 min of centrifugation at 4,000 rpm, supernatant (5 mL) was filtered through a 0.45 µm membrane filter (HVHP, Millipore). Subsequent clean up by strong anion exchange (SAX) solid phase extraction (SPE) was necessary to avoid tryptophan interference. Since DA can decompose when frozen, prepared SAX-cleaned extracts were analyzed immediately.

### 3.3. HPLC analysis

**Figure 7:**
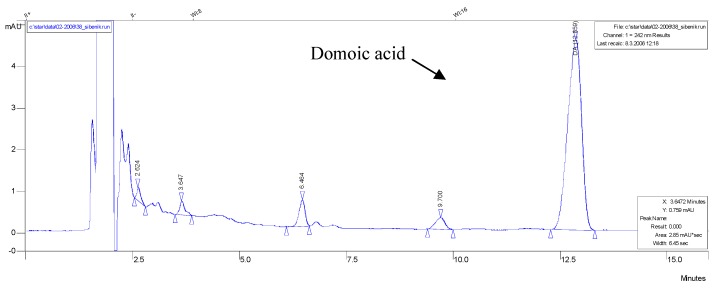
HPLC-UV chromatogram of contaminated mussel SAX-cleaned extract contains 6.5486 µg g^-1^ DA, retention time (DA) = 12.859 min.

The HPLC system consisted of a Varian ProSTAR 230 Solvent Delivery Module, 310 UV/Vis Detector, 335 Photodiode Array Detector and 410 Autosampler. The column used was a Pinnacle II C18, 250 × 4.6 mm (Restek), with a C18 Guard Cartridge (20 × 4 mm), at the temperature of 40 ºC. Domoic acid was detected by UV absorption at a wavelength of 242 nm, the DA absorption maximum. The mobile phase consisted of 100 mL acetonitrile, 0.2 mL trifluoroacetic acid and up to 1000 mL deionised water. Domoic acid has a retention time of around 12.8 min. Figure 7 shows a typical chromatogram of DA in mussel SAX-cleaned extract.

A standard calibration curve was performed by using an externally DA certified calibration solution for domoic acid (National Research Council of Canada, Halifax, NS, Canada), prepared in seven different concentrations (0.25, 0.5, 1.0, 2.5, 5.0, 10.0 and 25.0 µg mL^-1^) and measured in triplicate by HPLC.

**Figure 8:**
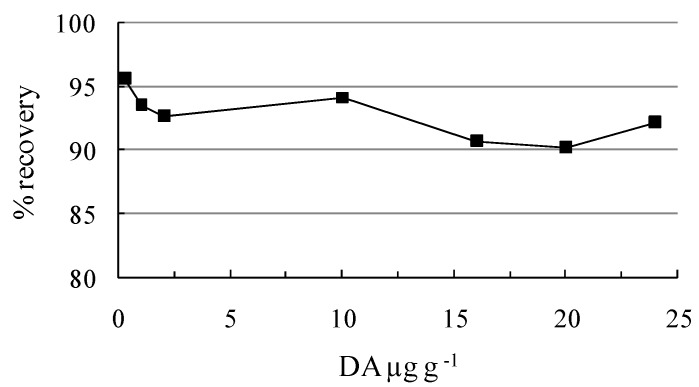
Recovery of DA in mussel tissues spiked at different levels using sax clean-up, n = 5.

Calibration curves were always linear, with correlation coefficients greater than 0.99. To establish an acceptable performance of the extraction procedure used, DA recovery was tested over an expected range of concentrations (Figure 8).

**Table 3:** Domoic acid concentrations in certified referent material ASP-Mus-c, n = 6.

Certified Concentrations (µg g^-1^)	Amount found (µg g^-1^)	Coefficient of variation (%)	Recovery (%)
41 ± 2	40.76	0.13	99.40
41 ± 2	40.61	0.10	99.05
41 ± 2	40.27	0.23	98.23
41 ± 2	39.42	0.74	96.14
41 ± 2	39.91	0.32	97.34

The accuracy of the procedure was determined by analysis of certified reference material ASP-Mus-c (National Research Council of Canada, Halifax, NS, Canada) and expressed as a ratio between the concentration found and the accepted reference concentration (Table 3). Limit of detection (LOD) was determined based on a generally accepted 3:1 signal-to-noise ratio. It was performed firstly by comparing the measured signal from samples with known low concentrations of DA to those of blank samples and establishing the minimum concentration at which the DA can be reliably detected. The limit of detection was found to be 0.1025 µg of DA per g of shellfish tissue. Secondly, it was determined by comparing the measured signal from certified calibration solutions with known low concentrations of DA to those of a blank solution and establishing the minimum concentration at which the DA can be reliably detected; the limit of detection of this method was 0.0220 µg mL^-1^.

### 3.4. Phytoplankton, temperature and salinity water sample analysis

Water samples for physical parameters and phytoplankton composition were collected at the same time as samples of shellfish taken at the stations shown in Figure 6. Phytoplankton samples were collected with a PVC sampler at the integrated depths from the surface to 5 m. The PVC line was pulled to close its base with a rubber stopper from the surface until the desired depth or the near-bottom of the water column. The collected water samples were fixed with glutaraldehyde solution (final concentration of 0.5%) and phytoplankton species were identified and counted under inverted microscope (Olympus IX50, Tokyo, Japan) according to Utermöhl [47]. Temperature and salinity of seawater was recorded by a CTD YSI63 probe.

## 4. Conclusions

The ASP investigation of shellfish from both Croatian breeding and harvesting areas revealed a negligible presence of DA over a wide Croatian Adriatic Sea area during the 2006 – 2008 period and all samples showing the presence of the toxin were found to have concentration levels of less than 20 µg g^-1^. This is the first detection of domoic acid in shellfish from Croatian waters, specifically from the central Adriatic Sea and estuarine waters (January, 2006). Toxin presence may be related to the identified *Pseudo-nitzschia* spp. blooms. Maximum levels of domoic acid detected in Croatian shellfish were found to be 6.5486 µg g^-1^, with a detection limit of 0.1025 µg g^-1^. Retention of DA in mussel, according to this study, is more than 42 days. It should be noted that there have been no DA poisoning outbreaks in Croatia to this date. *Pseudo-nitzschia* spp. bloom formation coincides with the temperature and salinity minima that occur in February. This research contributes to the knowledge of the interactions of *Pseudo-nitzschia* spp. and abiotic parameters at regional and global scales for the prediction and prevention of its harmful effects and potential toxicity. It will also contribute to the understanding of the occurrence of ASP in shellfish, a known member of the marine food web able to accumulate DA.
